# Diagnosis and treatment of COVID-19 complicated with liver abscess: A case report and literature review

**DOI:** 10.1097/MD.0000000000040839

**Published:** 2025-01-17

**Authors:** Fan Li, Xueqiang Jiang, Yanwei Liu, Wan Wang, Congyu Zhang

**Affiliations:** a Department of General Practice, Sinopharm Dongfeng General Hospital, Hubei University of Medicine, Shiyan, China; b Department of Infectious Diseases, Sinopharm Dongfeng General Hospital, Hubei University of Medicine, Shiyan, China.

**Keywords:** case report, COVID-19, diagnosis, liver abscess, treatment

## Abstract

**Rationale:**

Novel coronavirus pneumonia is spreading in many countries and regions. Coronavirus disease (COVID-19) is characterized by rapid onset, high infectivity, rapid progression, and variably effective treatment.

**Patient concerns:**

One 63-year-old woman presented to the fever clinic of our hospital on February 10, 2020, due to a fever for 12 days.

**Diagnoses:**

COVID-19 complicated with liver abscess.

**Interventions:**

A case report of a patient with COVID-19 complicated with liver abscess admitted to our hospital was used to explore the treatment methods for COVID-19 complicated with bacterial liver abscess. The sepsis caused by pulmonary viral infection and liver bacterial infection were correctly distinguished, and the patient was cured and discharged after targeted treatment, abscess, and pleural puncture and drainage.

**Outcomes:**

The patient was cured with a normal temperature is >3 days and coronavirus 3 consecutive negative nucleic acid detection.

**Lessons:**

COVID-19, elderly critically ill patients may be prone to rapid onset, complex disease, multiple organ damage, prolonged hospital stay, and high mortality. In the treatment of such COVID-19 patients, holistic thinking is required, pathology may not be limited to the lung, but may affect other organs, and treatment guidelines should not be blindly followed. Medication may need to be individualized for patients with COVID-19 complicated with liver abscess.

## 1. Introduction

At present, Novel Coronavirus Pneumonia (NCP)^[1]^ is spreading in many countries and regions. COVID-19 is characterized by rapid onset, high infectivity, rapid progression and variably effective treatment. Our hospital is a designated hospital for COVID-19 treatment in Shiyan City, receiving and treating suspected, mild, ordinary, severe and critical patients. In the course of a pandemic, a patient with severe fever and shock as the main clinical manifestation of COVID-19 complicated by liver abscess was admitted to our department. After literature review, no case of this type has been reported so far, which is now shared as follows:

## 2. Case presentation

### 2.1. Clinical data

The patient was a retired female aged 63 years, from Maojian District, Shiyan City, Hubei Province. She came to the fever Clinic of our hospital on February 10, 2020 due to “fever for 12 days.” She denied travel to an affected area or close contact with a COVID-19 patient. Her past medical history was unremarkable. The patient had fever without obvious cause on January 29, 2020, with the highest temperature of 39 °C, accompanied by fatigue and loss of appetite, without cold or chills, without cough, sputum, palpitations, chest tightness, asthma dyspnea, nausea, vomiting, abdominal pain, diarrhea, urinary frequency, urgency, dizziness or sweating. After a self-administered antipyretic diclofenac sodium suppository 50 mg, body temperature decreased, but symptoms recurred with intermittent fever, accompanied by tachypnea. Chest computed tomography (CT) examination, at another hospital, suggested bilateral lung infection and raised the possibility of viral pneumonia, therefore novel coronavirus nucleic acid test was performed in the fever clinic of our hospital and was found to be positive. Physical examination: Body temperature:36.7 °C (after diclofenac sodium 50 mg rectally), pulse:132/min regular, respiration:22/min, blood pressure:80/64 mm Hg, clear consciousness, red throat with congestion, red tonsils, coarse breath sounds in both lungs, no dry or wet rales. Abdominal examination showed no obvious abnormality, there was no edema of lower limbs and negative pathological signs.

### 2.2. Auxiliary examinations

**Figure 1.**
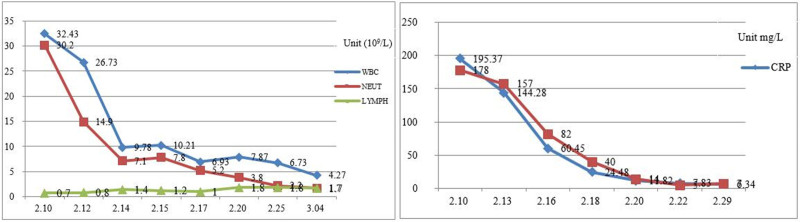
Changes in WBC, NBUT neutrophils (NEUT), LYMPH and CRP (February 10–March 4). CRP = C-reaction protein, LYMPH = lymphocyte, WBC = white blood cell.

**Figure 2.**
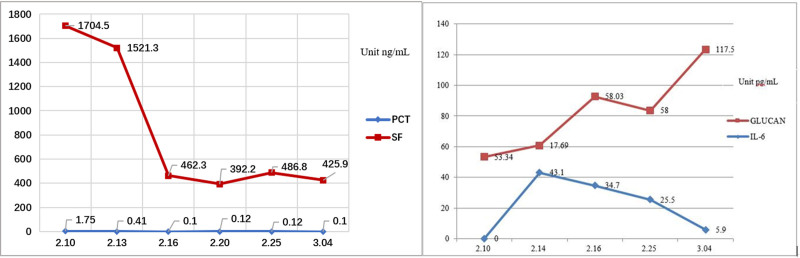
Changes in ferritin, PCT, SF, interleukin-6 and Polysaccharide (GLUCAN) (February 10–March 4). PCT = procalcitonin, SF = serum ferritin.

**Figure 3.**
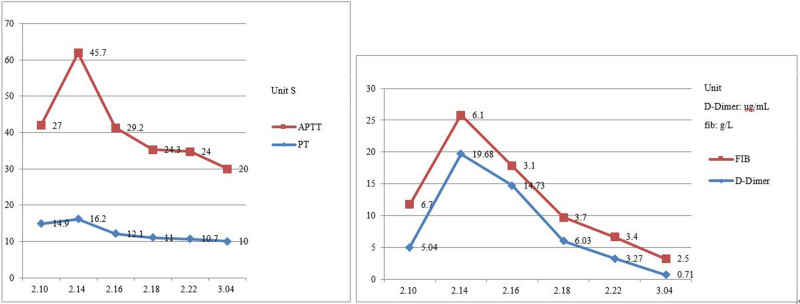
Changes in APTT, PT, FIB and D-dimer (February 10–March 4). APTT = activated partial thromboplastin time, FIB = fibrinogen, PT = prothrombin time.

**Figure 4.**
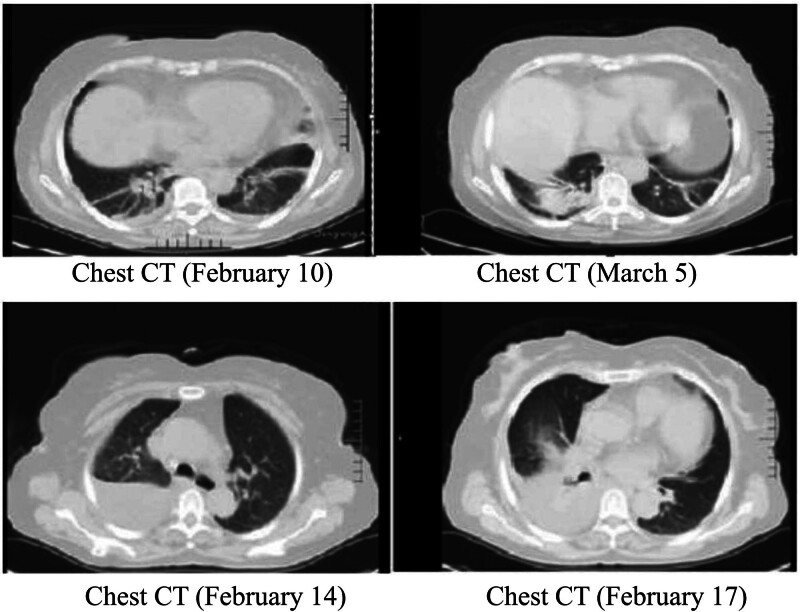
Chest CT. CT = computed tomography.

**Figure 5.**
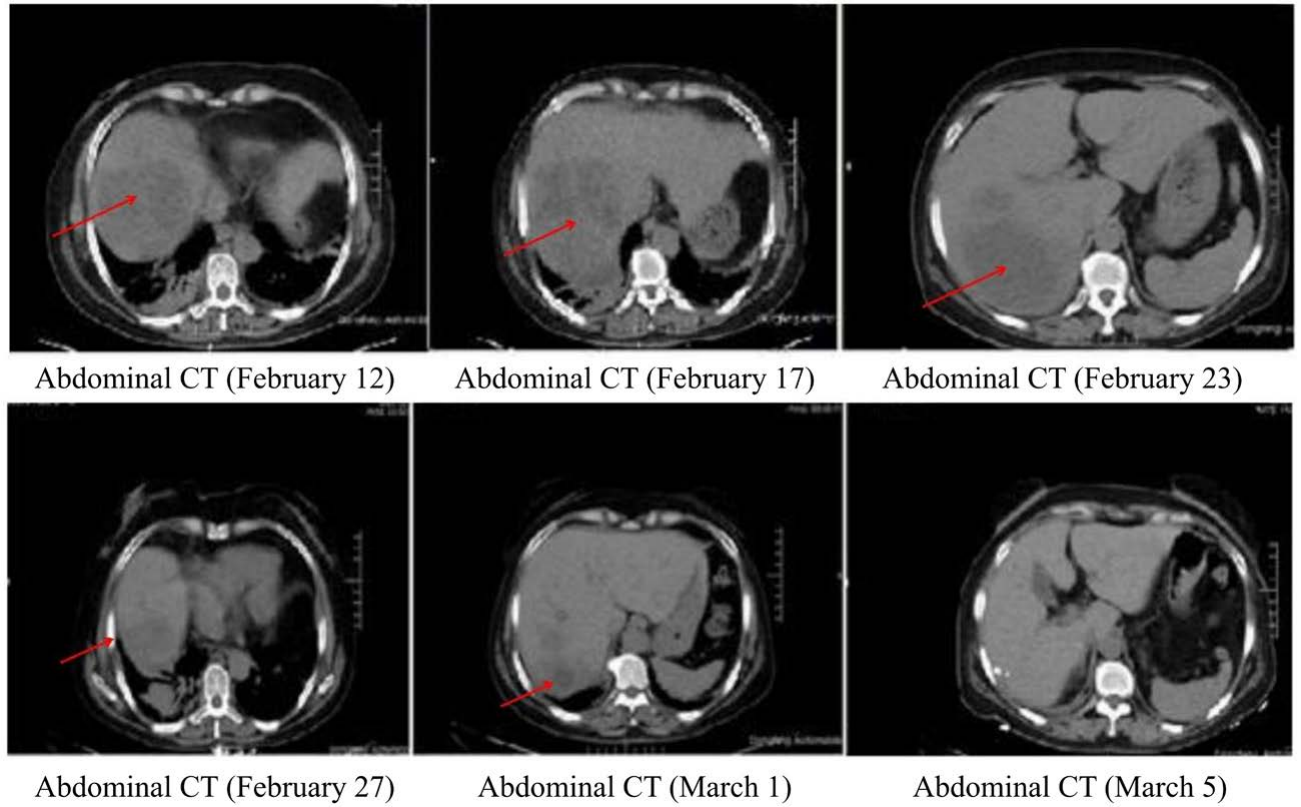
Abdominal CT. CT = computed tomography.

Blood gas analysis (on oxygen 5 L/min) suggested hypoxemia and respiratory distress, including pH: 7.499; the partial pressure of carbon dioxide: 35.2 mm Hg; the partial pressures of oxygen: 92 mm Hg; base excess: 4 mmol/L; hydrogen carbonate: 27.7 mmol/L; sulfur dioxide: 98%; Lactate: 1.51 mmol/L; oxygenation index: 224 mm Hg. Novel coronavirus nucleic acid test was positive on February 10, 2020, and was negative on February 15, 17, and 20, 2020, respectively. There were erythrocyte sedimentation and mild abnormal liver function. Blood analysis indicated that white blood cell was 32.71 × 10^9^/L, neutrophils was 30.2 × 10^9^/L, NEUT% was 92.3%, lymphocyte (LYMPH) was 0.7 × 10^9^/L, LYMPH% was 3.6%. C-reaction protein was 195.37 mg/L, serum ferritin was 1766.80 ng/mL, interleukin-6 was 146.5 pg/mL, procalcitonin was 1.75 ng/mL. Immunoglobulin M was weakly positive for influenza A + B virus, immunoglobulin M was weakly positive for parainfluenza virus types 1, 2, and 3. Blood culture was negative. There were multiple infectious lesions in both lungs on chest CT, indicating viral pneumonia in combination with the previous medical history. There were also bilateral pleural effusions, right larger than left, and slightly reduced hepatic density. Enhanced CT or magnetic resonance was recommended for further examination. Hepatobiliary pancreas and spleen ultrasonography suggested a possible large abscess in the right lobe of liver. Ultrasound of the thoracoabdominal cavity showed bilateral pleural effusions, with a large amount on the right side but no ascites. Drainage fluid culture of liver abscess showed *Klebsiella pneumoniae*. Five tumor markers: carcino-embryonic antigen, 0.9 ng/mL; alpha-fetoprotein, 5.46 ng/mL; serum ferritin, 1938.80 ng/mL; Ca125, 1558.70 U/mL; squamous cell carcinoma antigen, 15.20 ng/mL. Pleural biochemistry: total protein, 53 g/L; alkaline phosphatase, 129 U/L; adenosine deaminase, 13 U/L; lactate dehydrogenase, 341 U/L; amylase, 30 U/L; rheumatoid factor, <1.00 KU/L; high-sensitivity C-reactive protein, 29.49 mg/L. Pleural fluid appearance: yellow, transparent, no clot, specific gravity of 1.028, qualitative protein (+), white blood cell count of 4.615 × 10^9^/L, mononuclear cells of 48%, multiple nuclear cell classification count of 52%. There was no bacterial growth in pleural effusion culture over 5 days. Electrocardiogram showed sinus tachycardia, with abnormal T waves, and myocardial ischemia was considered. Brain natriuretic peptide, myocardial markers, renal function, and blood glucose level were normal. Urine and fecal routine cultures, and 5 quantification of viral hepatitis B, combination of hepatitis, Novel Coronavirus antibody, and A/B antigen and A/B respiratory syncytial virus nucleic acid were all negative. Blood routine examination, infection combination, coagulation indices and chest and abdominal CT examination appearances are shown in Figures 1–5.

### 2.3. Diagnosis and treatment process

Medical treatment was according to novel Coronavirus Infection Pneumonia Treatment Scheme (Trial Edition 6),^[2]^ as the combination of clinical manifestations, Chest CT images and positive nucleic acid test results were diagnostic of COVID-19. The patient was admitted with typical manifestations of severe disease: high fever, oxygenation index of 224 mm Hg, rapid heart rate, and shock. The patient was seriously ill, requiring high flow oxygen (5 L/min by mask) and a multidisciplinary discussion (Medical, Infection, Respiratory, and Radiology Departments) was held to formulate rescue and a treatment plan. Management included rapid infusion of sodium chloride solution, and dextran 40 solution to combat shock, antibiotics: imipenem-cilastatin sodium injection, 1.0 gram (g) q8h, + vancomycin injection, 1.0 g q12h, later replaced by ceftazidime, 2.0 g q12h, Arpidol hydrochloride 0.2 g 3 times a day, and recombinant human interferon a-1b 5 million IU the antiviral by nebulization, vitamin C injection 4 g, Xuebijing injection 100 mL to reduce inflammation, Chinese medicine prescription granules, Thymus method 1.6 mg twice a week, spleen polypeptide by injection 6 mL to regulate immunity, 20% human serum albumin 100 mL for correction of low plasma proteins, reduced glutathione needle 2.4 g + isoglycyrrhizase needle 20 mL intravenously to protect liver enzymes, panxitora azole enteric capsules for prevention of stress ulceration, the bow for injection heparin calcium 1500 IU subcutaneously qd as anticoagulation, for prevention of thrombosis, correction of electrolyte disorders, lactulose oral liquid runchang purge, compound lactobacillus acidophilus + garlic soft capsule for adjustment of intestinal flora, inhaled acetylcysteine solution + suction cloth to Ned suspension liquid atomization inhalation as an expectorant, the antioxidant, ambroxol hydrochloride oral solution + Yumei capsules oral for cough and others supportive treatment. According to the results of chest and abdominal CT, bedside hepatobiliary and thoracic B-ultrasound, percutaneous liver puncture catheter drainage was performed for liver abscess on February 11, 2020, under the guidance of ultrasound. Brown abscess fluid, mixed with a small amount of hemorrhagic material was extracted, and drainage fluid culture + drug sensitivity was sent. About 600 mL of light red abscess fluid was drained the next day. A chest drain placed in the right thoracic cavity, under ultrasound guidance on February 18, 2020, drained 150 mL and pleural fluid was sent for routine examination, biochemical examination, swelling mark and culture. February 21, 2020, Chest and abdominal drainage bags and not drainage liquid, to root out the chest and abdominal cavity drainage tube, stable vital signs. Admitted to hospital after 23 days (March 4, 2020), patients with normal temperature is more than 3 days, will be coronavirus 3 consecutive negative nucleic acid detection, review the chest CT hint double lung disease stove did not see obvious change and liver abscess is absorbed before narrowing, chest, abdominal cavity no effusion on both sides. After discussion by Panel discussion, the patient was discharged from the hospital with isolation observation and follow-up.

### 2.4. Final diagnosis

COVID-19 severe pneumonia; Bacterial liver abscess; Septic shock; Pleural effusions; Influenza; Abnormal liver function; Electrolyte disturbance; and Hypoproteinemia.

## 3. Discussion

This 63-year-old woman with COVID-19 admitted to our hospital, denied any history of underlying disease, and her blood glucose monitoring was fair. Initial symptoms were fever, loss of appetite and fatigue, suggestive of viral infection, with positive SARS serology, but by 12 days she had developed bibasal pulmonary changes including small pleural effusions. In hospital she had remittent high fever and developed sinus tachycardia, hypoxemia, respiratory distress, hemodynamic instability, myocardial ischemic changes on electrocardiogram and shock. Small bilateral pleural effusions were present on admission, but they increased significantly over 4 days, with a large right pleural effusion appearing 1 week later. After admission, liver function was abnormal chest CT suggested a large low-density shadow in the right lobe of the liver. The possibility of a tumor or abscess was considered, but abscess was confirmed by bedside color ultrasound. Laboratory investigations of the pat suggested multi-system damage; progressive increase in D-dimer, abnormal liver function, markedly increased white blood cell, neutrophil, and platelet counts, progressive elevation of ferritin and procalcitonin, prolonged prothrombin time and activated partial thromboplastin time. *K pneumoniae* was cultured from the liver abscess from sepsis, although no bacterial growth was observed in 2 blood cultures. Sepsis could still be diagnosed from the clinical manifestations.

Because the patient had no obvious respiratory symptoms at the onset of fever, we consider it likely that she initially presented with COVID-19 and subsequently developed a liver abscess and sepsis. We consider it less likely that a bacterial pneumonia was the primary condition because of the imaging and clinical sequence. Although pleural effusion occurs relatively rarely in COVID-19 (in 5.6% of CT scans,^[3]^ it was reported in severe cases in some elderly patients with underlying diseases, although the mechanism was unclear^[4]^). In our patient, other causes including cancer, tuberculosis, autoimmune, cardiac and renal were ruled out; pleural effusion was an exudate with typically elevated protein, lactate dehydrogenase and inflammatory cells and it was likely related to the liver abscess.

The presentation with COVID-19 and development of respiratory failure would normally indicate glucocorticoid treatment, however, this was withheld in the face of liver abscesses and bacterial infection.

We have found only 3 case reports of bacterial liver abscess being diagnosed in patients with recent COVID-19,^[5–7]^ an amebic liver abscess^[8]^ and a 36-year-old man with a liver abscess who was SARS-COV 2 positive.^[8]^ The bacterial liver abscesses all occurred 4 to 6 weeks after initial COVID-19; 1 patient had received dexamethasone^[6]^ and 2 tocilizumab.^[5,7]^ Interestingly, *K pneumoniae* was isolated from blood and liver aspirate in a 78-year-old man 6 weeks after admission with severe COVID-19 pneumonia.^[9]^ Our patient appears unique in presenting with a liver abscess early into the illness and before receiving any immunosuppressive therapy. We present this case to highlight the importance of considering additional diagnoses in a patient with COVID-19 and to stress the importance of individualizing the treatment strategy (here withholding corticosteroid therapy) in accordance with the patients’ overall clinical picture.

## Acknowledgments

The authors would like to express their gratitude to Edit Springs (https://www.editsprings.cn) for the expert linguistic services provided.

## Author contributions

**Conceptualization:** Xueqiang Jiang.

**Formal analysis:** Xueqiang Jiang, Yanwei Liu, Wan Wang, Congyu Zhang.

**Funding acquisition:** Xueqiang Jiang, Wan Wang.

**Investigation:** Fan Li.

**Methodology:** Congyu Zhang.

**Project administration:** Fan Li, Wan Wang.

**Resources:** Yanwei Liu.

**Validation:** Fan Li, Congyu Zhang.

**Visualization:** Yanwei Liu.

**Writing – original draft:** Fan Li, Xueqiang Jiang, Wan Wang.

**Writing – review & editing:** Xueqiang Jiang, Wan Wang.
